# Single-cell omics traces the heterogeneity of prostate cancer cells and the tumor microenvironment

**DOI:** 10.1186/s11658-023-00450-z

**Published:** 2023-05-09

**Authors:** Xudong Yu, Ruijia Liu, Wenfeng Gao, Xuyun Wang, Yaosheng Zhang

**Affiliations:** 1 Dongzhimen Hospital, Beijing University of Chinese Medicine, Beijing, 100700 China; 2 Beijing Tumour Minimally Invasive Medical Center of Integrated Traditional Chinese and Western Medicine, Beijing, 101121 China; 3 Beijing Hospital of Traditional Chinese Medicine, Capital Medical University, Beijing, 100010 China

**Keywords:** Prostate cancer, Single-cell omics, Spatial transcriptomics, Heterogeneity

## Abstract

Prostate cancer is one of the more heterogeneous tumour types. In recent years, with the rapid development of single-cell sequencing and spatial transcriptome technologies, researchers have gained a more intuitive and comprehensive understanding of the heterogeneity of prostate cancer. Tumour-associated epithelial cells; cancer-associated fibroblasts; the complexity of the immune microenvironment, and the heterogeneity of the spatial distribution of tumour cells and other cancer-promoting molecules play a crucial role in the growth, invasion, and metastasis of prostate cancer. Single-cell multi-omics biotechnology, especially single-cell transcriptome sequencing, reveals the expression level of single cells with higher resolution and finely dissects the molecular characteristics of different tumour cells. We reviewed the recent literature on prostate cancer cells, focusing on single-cell RNA sequencing. And we analysed the heterogeneity and spatial distribution differences of different tumour cell types. We discussed the impact of novel single-cell omics technologies, such as rich omics exploration strategies, multi-omics joint analysis modes, and deep learning models, on future prostate cancer research. In this review, we have constructed a comprehensive catalogue of single-cell omics studies in prostate cancer. This article aimed to provide a more thorough understanding of the diagnosis and treatment of prostate cancer. We summarised and proposed several key issues and directions on applying single-cell multi-omics and spatial transcriptomics to understand the heterogeneity of prostate cancer. Finally, we discussed single-cell omics trends and future directions in prostate cancer.

## Introduction

According to the global Cancer data in 2020 released by the International Agency for Research on Cancer (IARC), Prostate cancer (PCa) is still the second most common cancer in men worldwide. Also, PCa is one of the main reasons for cancer-related deaths in men worldwide [1]. Cancer prediction data released by the American Cancer Society (ACS) show that by 2022, there will be about 260,000 new PCa cases in the new cancer cases in the United States, accounting for 27% of all cancer cases in men [2].In the formation of prostate cancer cells, gene mutations in normal epithelial cells are the primary way to induce PCa. However, the factors driving PCa progression are complex, and it is not enough to explore the causes of the cancer cells themselves [3, 4]. Previous studies have shown that the interaction between malignant epithelial cells and tumour microenvironment (TME) is a critical cause driving the progression of PCa [5, 6]. PCa progression is also a complex process that coordinates crosstalk between tumour cells and TME components [7]. Tumour cells can change and maintain their survival and development conditions through autocrine and paracrine, promoting cancer development and progression [8, 9]. In PCa, crosstalk between some components of TME promotes the malignant proliferation of tumour cells.

**Fig. 1:**
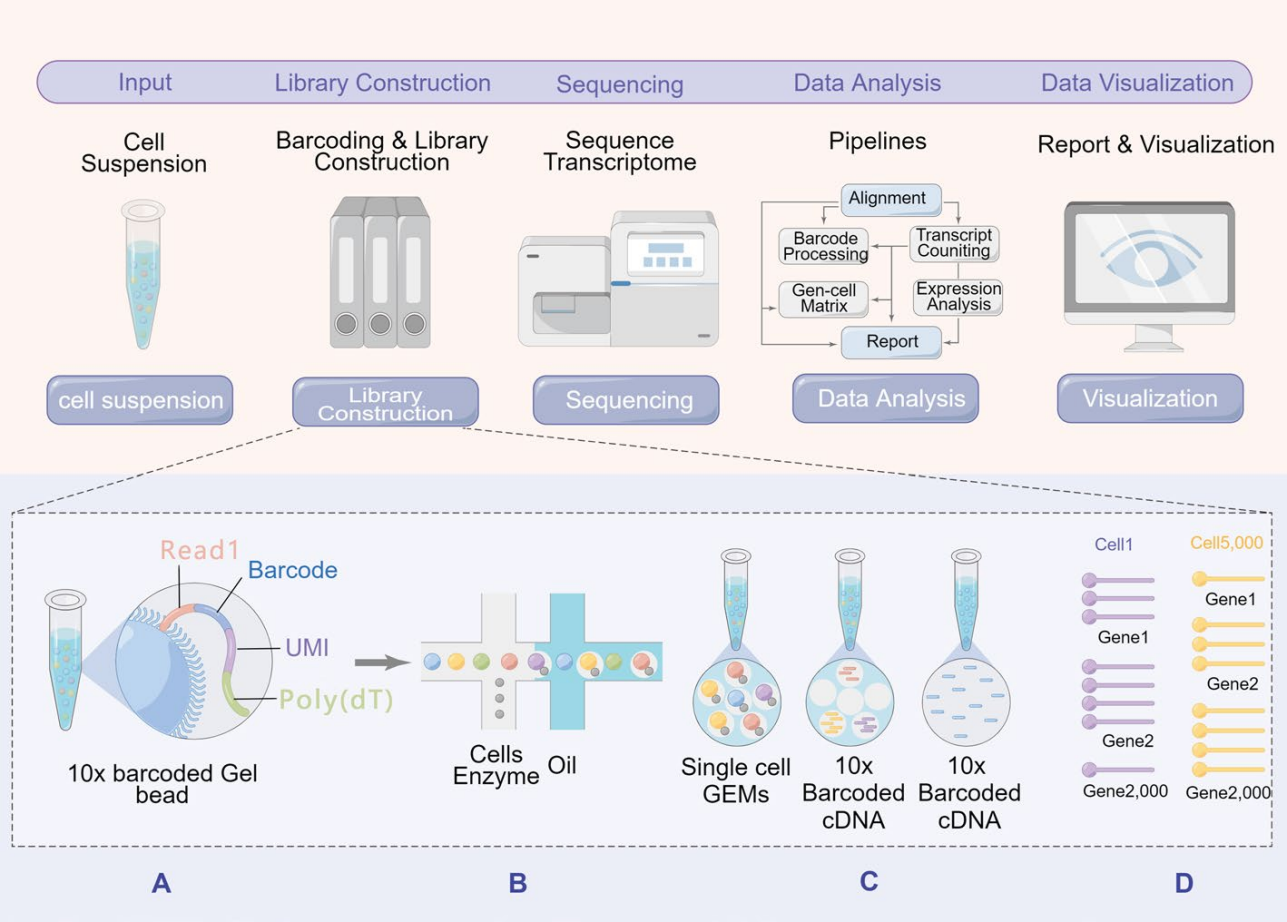
Single-cell sequencing flow chart. **A** Read 1 is derived from beads and carries the sequence of the RNA molecule; Barcode is used to distinguish between cell types; UMI is used to distinguish between RNAs; a UMI is added to each RNA, thus ensuring that the same RNA molecule carries the same UMI after amplification; Poly(dT) on the beads binds to the poly A tail at the end of the RNA. **B** The cell suspension, beads and oil droplets are added at three locations; when the cell suspension enters, it combines with the beads; after the combination of the two, they are wrapped by the oil droplets, forming an oil-in-water structure. **C** 10×Barcoded Gel Beads are mixed with cells, enzyme, and partitioning oil. Single cell GEMS undergo reverse transcriptase to generate 10× Barcoded cDNA. All generated cDNA from individual cells shares a common 10× Barcode. **D** The resulting 10x Barcoded library can be directly used for single cell whole transcriptome sequencing or targeted sequencing workflows

Traditional research methods are all aimed at specific cell populations. However, PCa is a tumour type characterised by high heterogeneity. Immunohistochemistry (IHC), immunofluorescence and other experimental methods are challenging to identify and analyse highly heterogeneous PCa. Therefore, it is difficult to provide complete information about tumour cells by traditional research methods alone. In recent years, the rapid development of single-cell omics has allowed us to understand the changes in a cell population, biochemical characteristics, and immune status of tumour tissues during disease progression [10]. In addition, single-cell RNA-sequencing (scRNA-seq) combined with various immunofluorescence techniques can analyze tumour cells at multiple levels and perspectives. As a result, single-cell omics help elucidate the molecular mechanism of PCa on the occurrence, development, metastasis, drug resistance and immune escape of tumour cells. Therefore, the review summarized the research status of PCa from the perspective of single-cell omics. The workflow for scRNA-seq is shown in Fig. 1. In conclusion, we summarized and presented several critical directions in applying single-cell multi-omics and spatial transcriptomics to understand the heterogeneity of PCa. We hope our work can provide some reference for the future research direction of PCa.

## Heterogeneity of TAECs in PCa

Prostate tissue comprises various cell types, including epithelial, stromal, and immune cells. Each cell type has its distinct gene expression profile [11]. During the progression of normal prostate tissue to PCa tissue, the complex interaction between tumour cells and surrounding epithelial cells and stromal cells has been widely studied [12, 13]. Previous studies have found that prostatic epithelial cells mainly include secretory epithelial cells, basal epithelial cells, luminal epithelial cells and neuroendocrine cells [14–16]. Transformation of the carcinogenic status of prostate epithelial cells during tumour progression mainly includes alteration of luminal epithelial cells and loss of basal epithelial cells. Single-cell sequencing of PCa tissue samples identified different cell types. These cells included epithelial cells, stromal cells (endothelial cells, fibroblasts, and smooth muscle cells), immune cells (T cells, bone marrow) cells, plasmocytes, mast cells, and B cells). “Epithelial cells” constituted the largest population of PCa cell types. With the wide application of single-cell omics, the deeper heterogeneity of tumour-associated epithelial cells (TAECs) in the development and progression of PCa has been gradually discovered. For example, Henry et al. [17] analysed cell profiles of the prostate and prostatic urethra by scRNA-seq and flow cytometry. They identified two new epithelial cell types, hillock cells and club cells, in prostate tissue. It may be associated with progression to different kinds of PCa.

Hanbing Song and colleagues [18] analysed cell states associated with tumourigenesis in PCa samples by single-cell omics. They performed a comprehensive cell-level analysis of epithelial cell subsets, stromal cells, and TME by scRNA-seq. It was found that ERG-tumour cells exhibit common heterogeneity with surrounding luminal epithelial cells compared to ERG + cells and may cause a typical TME response. Genetic profiling by single-cell omics indicated that PCa-club cells have a highly androgen-responsive state. In addition, there was heterogeneity in prostate epithelial cells marked by high androgen signalling state and enriched in PCa with LTFhigh and NKX3-1high luminal-like cell state. Another study identified gene expression profiles of epithelial cell populations from single-cell data and bulk RNA-seq data from human prostate tissue samples. It confirmed the presence of luminal, basal, or bipotential progenitor populations [17]. These progenitor populations have specific anatomical locations and are potentially associated with different phenotypes of PCa [19, 20]. Hyunho Han and colleagues [21] performed a secondary analysis of these data and defined these prostate epithelial cell genes into four tumour clusters. Furthermore, they performed CIBERSORT deconvolution and found that luminal subtypes over-expressed Aminopeptidase N (CD13) for ANPEP and relaxin for RLN1. They were all expressed explicitly by mature luminal epithelial cells. In addition, epithelial cells with different subtypes of PCa and multiple subtypes of tumour-associated epithelial cells have been identified in breast cancer. Another study [22] performed scRNA-seq on 14 untreated PCa patients. The study identified four tumour cell transcriptional isoforms: EMT-like (subtype 0), luminal A-like (subtype 1), luminal B/C-like (subtype 2), and basal-like (subtype 3). Furthermore, these four transcriptional isoforms corresponded to distinct tumour subclonal patterns. Their study provided an analysis of subclones and transcriptional heterogeneity and their impact on the prognosis of PCa patients. In addition, scRNA-seq was used to analyse the transcriptome of more than 20,000 primary human breast epithelial cells isolated from mammaplasty surgeries in seven individuals [23]. Secretory L1 cells and hormone-responsive L2 cells were identified. The diversity of these breast epithelial cells lays the foundation for the progression to breast cancer. They may also serve as the cells of origin for different breast cancer subtypes.

The above studies all suggest that the heterogeneity of TAECs is involved in the malignant progression of tumours in multiple ways. Moreover, androgen hyperresponsiveness is exhibited chiefly in PCa, which further induces the progression to castration-resistant prostate cancer (CRPC) and is associated with endocrine therapy resistance.

## CAFs function subclusters in PCa

Cancer-associated fibroblasts (CAFs) are the main components of the solid tumours microenvironment, accounting for more than 50% of tumour stromal cells [24, 25]. CAFs are the centres of cross-communication between various cells in the tumour stroma. CAFs are highly heterogeneous in terms of phenotype, origin, and function. This heterogeneity of CAFs is essential in developing and progressing various tumours, including promoting cancer cell growth, angiogenesis, extracellular matrix (ECM) remodelling, and immunosuppression [26, 27]. In recent years, the identification of subpopulations of CAFs has been completed by different experimental techniques such as immunohistochemistry, situ hybridisation, flow cytometry and fluorescence-activated cell sorting (FACS). However, the initial information and cellular origin of CAFs subsets still need to be clarified [28]. The advent of scRNA-seq has dramatically changed the field of study of CAFs and revealed additional complexities.

The first role of single-cell histology is to explore the heterogeneity of CAFs, that is, to classify subpopulations of CAFs by scRNA-seq. Bartoschek et al. [29] applied scRNA-seq to identify three subgroups of CAFs in breast cancer: vascular CAFs (vCAFs), matrix CAFs (mCAFs) and developmental CAFs (dCAFs). Each of the three subgroups of CAFs performs a different cellular function. Matrix CAFs can produce a diversity of matrix components in large quantities. However, vascular CAFs and developmental CAFs specialise in producing basement membrane products and paracrine signalling molecules, respectively [29]. An analysis of scRNA-seq data also detected two different cell clusters of CAFs, namely “Fibroblasts” and “Myofibroblasts/Mural cells” [30]. A second important role of single-cell omics is the identification of differential genes and specific markers associated with CAFs. Han Luo et al. combined the single-cell public database and their scRNA-seq data for a pan-cancer analysis of 10 solid cancers [31]. This study found that angiogenesis and immunomodulation-related genes (e.g., PDGFRA, PDGFRB, FAP, NOTCH 3, HES 4, and THY 1) were significantly upregulated in CAFs. However, it has been possible to identify different subtypes and differential genes of CAFs at the resolution of single cells. However, no clear distinction between CAFs and normal fibroblasts can be made. It is generally clear that the significant CAFs are derived from normal fibroblasts and have evolved into different differentiation states that may affect TME.

In recent years, related studies have reported the application of single-cell omics for identifying CAFs in the PCa microenvironment. A study [32] probed the heterogeneity of CAFs in human primary prostate cancer by two different scRNA-seq methods (10 × Chromium and Fluidigm C1) and identified six subgroups of prostate cancer-associated fibroblasts (PCAFs). The results showed that PCAFs enhanced the recruitment of myeloid cells in vitro and in vivo and that monocyte migration was dependent on PCAFs-derived CCL2. Another study [33] performed a combined analysis of RNA-seq data and CHIP-seq data and showed that CCL2 and CXCL8 cytokines enhanced migration and invasion of PCa cells mediated in CAFs. Moreover, targeted blockade of CCL2 and CXCL8 cytokine expression was able to eliminate this cell migration and invasion. Similarly, the previous study had shown that CAFs induced cell migration of PCa [34]. The above findings indicated that CAFs could promote PCa progression through various cytokines.

In general, CAFs play an essential role in the progression of PCa. The fibroblasts were classified based on the similarity between each cell cluster and the expression of crucial marker genes. The results of the above studies also indicate that there is heterogeneity with CAFs in TME. CAFs enhance tumour cell colonisation by inducing tumour growth, invasion, epithelial-mesenchymal transition (EMT) and drug resistance. Therefore, CAFs identified by scRNA-seq may play an essential role in promoting the malignant progression of PCa.

## Single-cell omics analysis of TIME of PCa

**Fig. 2:**
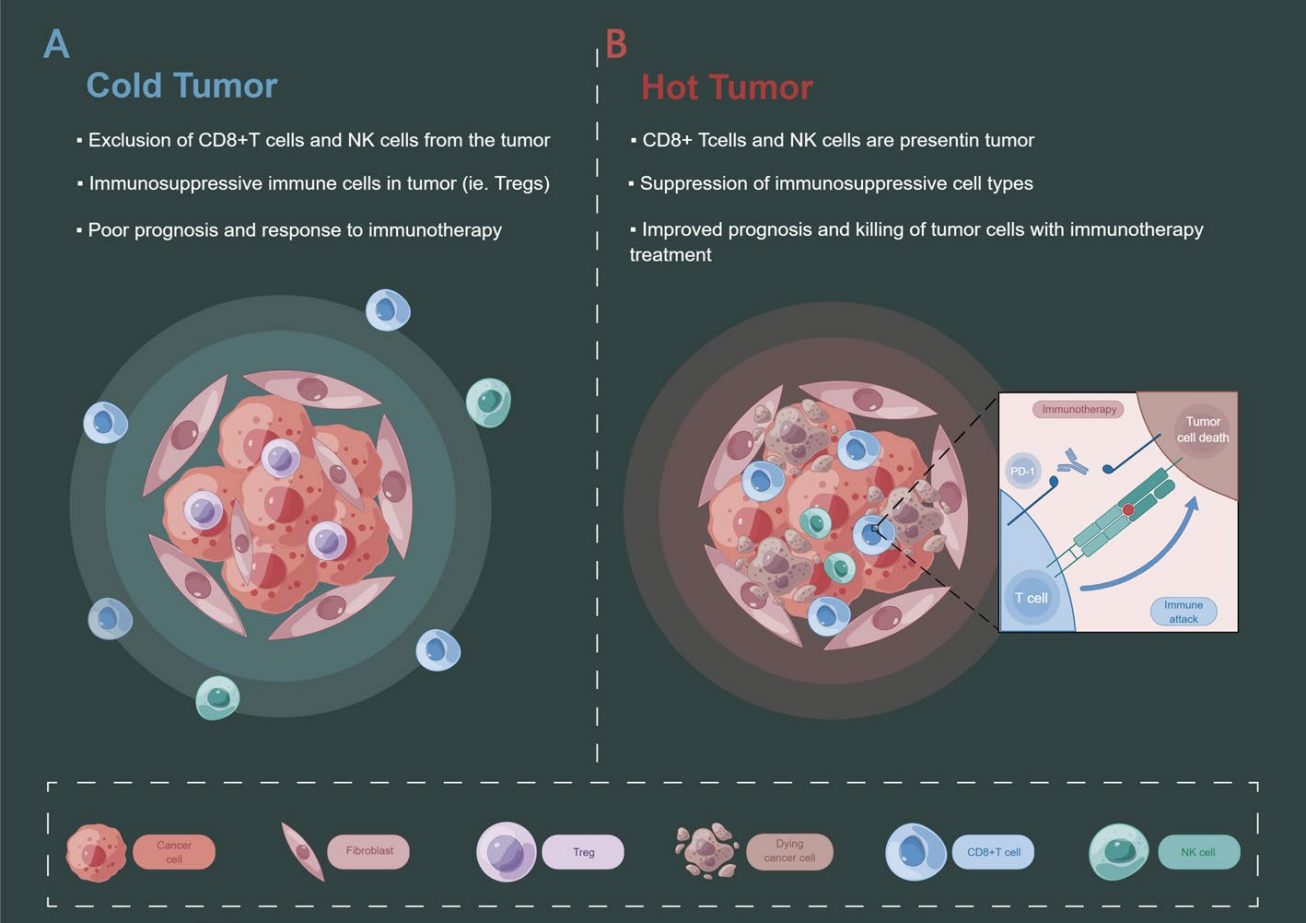
Cold tumour vs hot tumour. **A** A cold tumour is a kind of immune cell suppressive tumour with few immune cells inside and in the tumour microenvironment, or the immune cells are difficult to penetrate. **B** A hot tumour is an immune cell infiltrating tumour, there are many immune cells near the tumour tissue, and the antagonism between tumour cells and immune cells is more active than a cold tumour.

Tumour immune microenvironment (TIME) is a complex structure, including immune activation, immune suppression, the residence of immunity, immune rejection, and other subtypes. TIME involves various cell types such as lymphocytes (T lymphocytes, B lymphocytes, etc.), monocytes, macrophages, etc. [35]. In addition, TIME also includes immunomodulatory molecules, chemokines, etc. TIME is crucial in tumour development and drug resistance and can affect disease prognosis and outcome [36, 37]. Generally, higher immune infiltration predicts better immune control and prognosis [38, 39]. Many immune cells are near hot tumour tissue, and the antagonism between tumour cells and immune cells is more active [40]. However, the previous conventional understanding presented some contradictory results in the study of PCa [41, 42]. PCa is usually considered a “cold tumour” [43, 44]. Cold tumours are immunosuppressive tumours with fewer immune cells inside the tumour and in the TME, or immune cells are difficult to penetrate [40] (Fig. 2). This may also be one of the critical reasons for the transformation of PCa into CRPC. CRPC often does not respond well to immunotherapy [45–47]. The complexity of the immune microenvironment of PCa leads to some contradictory results with the standard “hot tumours”. For example, the study [48] showed that a high density of CD8 + T cells in the tumour region was associated with an increased risk of clinical progression in PCa patients. A study by Lecler and colleagues also found that a higher density of stromal CD8 + TIL was associated with poor prognosis in PC [49]. The above results suggest a complex regulatory mechanism between cells in the TIME of PCa. How to make the TIME of prostate change from “cold” to “hot” will be the highlight of future research [50–52]. Therefore, a more in-depth analysis of TIME in PCa at the single-cell level is necessary.

Single-cell omics has been an emerging technical field in recent years, and the number of studies on the TIME of single-cell omics in PCa is limited. We selected representative scRNA-seq applications in TIME for review. A study [53] integrating data from RNA-Seq and scRNA-seq synthesised the expression patterns of hub genes in different cell subsets of PCa. It delineated the characteristics of TIME and prognostic markers in PCa. Another study [54] performed a single-cell transcriptome analysis of more than 30,000 cells from 13 PCa samples. Multiple transcriptomic programs of TME were found to be activated, especially reprogramming involving TIME-related cells. The study noted that prostate cancer cells could alter the T cell transcriptome, which involves increased KLK3 abundance in T cells. In addition, macrophages are an integral component of TIME. Macrophages can inhibit the progression of cancer cells and can also promote further cancer progression. Joseph et al. [55] isolated myeloid cells from post-PCa resection specimens by single-cell transcriptome sequencing and identified a unique macrophage population in PCa. This study characterised a novel phenotype of PCA-associated macrophages and identified the colony-stimulating factor 1 receptor (CSF1R) as a critical regulator of immunosuppressive macrophage expansion. Several of the above studies illustrate the unique advantages of single-cell omics in studying the heterogeneity of TIME and provide a powerful platform for dissecting the complexity and heterogeneity of TIME and discovering new biomarkers.

## Heterogeneity of the spatial distribution of PCa

### "Microscopic heterogeneity" of the spatial distribution of PCa

Single-cell omics provide a new perspective for the development and evolution of tumours [56, 57]. However, this method breaks the complete structure of the tissue in the process of dissociating each cell, which means that the spatial distribution and pathological information of the cells are lost [58, 59]. Therefore, researchers have developed various technologies and methods for spatial transcriptome analysis combined with scRNA-seq, such as slide-seq and high-definition Spatial Transcriptomics (HDST) [60, 61].In addition, Keren et al. [62] also applied multiplexed ion beam imaging by time-of-flight (MIBI-TOF) to describe the spatial information of tumour cells.

Studies have shown that PCa patients with Gleason score greater than 7 presented higher heterogeneity and are more likely to progress to CRPC [63, 64]. Currently, several whole-genome and transcriptome sequencing studies on PCa have been completed. These studies provide a comprehensive genomic database of PCa and reveal associated somatic mutations and Copy number variation (CNV) [65–67]. However, they inevitably lack the spatial information of PCa transcriptome genes. For the first time, Berglund et al. [68] measured spatial gene expression in at least 6000 regions of prostate cancer tissue. Significant heterogeneity was found between different spatial transcriptomes of the same tumour sample. Moreover, the heterogeneity can be used as a basis for early clinical evaluation of PCa. A detailed spatial sampling of 23 different tumour regions was performed in one study to assess the heterogeneity of prostate cancer cells at the genomic level within different lesion regions [69]. No CNVs and very few single-nucleotide variants were shared between PCa lesions. This result provides evidence for the existence of polyclonality in PCa. The spatial transcriptome information of PCa also indicates that tumour cells are highly heterogeneous regarding genomic rearrangements. In addition, a recent spatial transcriptomics study [70] analysed different clonal patterns in PCa tumour tissues and adjacent benign tissues. This study found that benign cells do not undergo phenotypic transition immediately after acquiring mutation burden, and there is an intermediate “metabolically active state”. Hence, CNV is an early event in cancer development. Another study [71] explored the spatial structure information of PCa by combining laser catapulting and single-cell DNA sequencing. The researchers found that tumour cells at different locations in PCa had different mutation patterns. It also implicated that PCa immune escape may be related to tumour cell spatial distribution heterogeneity. Since spatial transcriptomics is still in its infancy, there are some technical biases in the research process. After accumulating enough spatial transcriptome models, researchers are expected to accurately identify tumour subtypes in the spatial dimension and make more precise therapeutic interventions.

### "Macroscopic heterogeneity" of the spatial distribution of PCa

Modern medicine divides the prostate into three main zones: peripheral zone (PZ), transition zone (TZ) and central zone (CZ) [72]. The theory of prostate zones has been widely applied and demonstrated since it was proposed. The differences in the pathogenesis, imaging features, histological features, biological behaviour and malignant potential among different prostate zones have been gradually revealed [73, 74]. An exciting finding in the regional spatial distribution of PCa is the significant variability in the incidence of prostate cancer in different zones (PZ, TZ and CZ). Many pathological morphologies have confirmed that PCa and prostatitis are primarily in the PZ. At the same time, benign prostatic hyperplasia is almost always found in the TZ, and the CZ is rarely involved in cancer or hyperplasia. Different prostate regions also have variability in biological behaviour and malignant potential [75, 76]. Studies have shown that PCa in PZ has higher malignant potential and worse clinical outcomes than TZ [77]. Studies have shown that PCa originating from TZ has a lower probability of seminal vesicle infiltration, extracapsular extension, and lymph node involvement [78]. In addition, few tumours have been reported to occur in prostate CZ. The only relevant literature reports show that PCa with CZ has the worst clinical outcome [79, 80].

A recent study [81] identified PZ and TZ cell types in older men’s prostate tissue by scRNA-seq. The results show that TZ aggregates more club and hillock cells than PZ. Moreover, PZ contained more TFF3 + cells than TZ. However, the KLK3 + and IDH1 + 4 subcluster luminal cells were more enriched in TZ. The expression of Notch pathway receptors (Notch1 and Notch2) and notch signal transduction activity were significantly increased in club and hillock cells. Notch signalling is a driving force in regulating stem or progenitor cell biology in various tissues [82, 83]. Various rare progenitor cells have been identified in prostate tissue and be associated with the origin of PCa [73, 74, 84, 85]. It also suggests a stronger Notch pathway activity in the TZ region of the prostate. In addition to different cell types and subsets, multiple genes are differentially expressed between different prostate zones. The study has shown that differentially expressed genes between TZ and PZ persist in PCa from similar regions and correlate with Gleason scores [86]. PZ and TZ of prostate tissue primary differences gene: BMP5, KIAA1210, TSPAN8, FOLH1B, TBX4, FOLH1, LAMA2, CPA3, FAM3B, CDH26 and TFPI. Regarding the above differential genes, it has been shown that BMP5 is a regulator of PCa progenitor cells and may be involved in the development of bone metastasis in cancer cells [87]. In addition, the study by Sakai and colleagues [88] found that the expression of Ki-67, MMP-2, and MMP-9 in PCa of PZ was significantly higher than TZ. Another study [89] evaluated cell proliferation and apoptosis in PCa of TZ and PZ, which showed similar apoptosis rates, but significantly higher cell proliferation rates in PZ than in TZ. P53 and bcl-2 were more frequently expressed in PCa of PZ than TZ. High expression of Ki-67 and bcl-2 genes correlates with the invasive potential of tumour cells [90, 91]. It provides a possible biological basis for PCa cells in PZ being more prone to extra-prostatic spread than those in TZ.

The biological differences of PCa between TZ and PZ suggest that tumour cells in different spatial regions have different pathogenic pathways. Therefore, identification and analysis of cellular and molecular differences in different spatial areas of the prostate can help reveal specific risk factors between different zones. Meanwhile, the region of origin of tumour cells should be considered an essential factor in the study of diagnostic and prognostic biomarkers of PCa. In this regard, single-cell omics and spatial transcriptomics highlight significant advantages.

## Identification of PCa-associated senescent cells

Cellular senescence (or merely ‘senescence’) is a specific, irreversible anti-proliferative mechanism that protects against tissue homeostasis. The process of cellular senescence acts as a complementary mechanism to programmed cell death, enabling the inactivation of diseased, dysfunctional, or nonessential cells at the appropriate time. Preceding studies [92, 93] have shown a positive protective effect of cellular senescence in limiting the malignant progression of tumours. However, in recent years, evidence suggests that senescent cells also stimulate tumour initiation and progression in different ways under certain circumstances [94–96].

The unique mechanism of cellular senescence in PCa has been reported several times. Exposure of PCa to different anticancer compounds, ionizing radiation, and selected AR ligands induces a senescence phenotype known as therapy-induced senescence (TIS). The most common cellular TIS signaling pathways in PCa are p53/p21^WAF1/CIP1^, p15^INK4B^ /p16^INK4A^/ pRb/E2F/Cyclin D, ROS/ERK, p27^Kip1^/CDK/pRb and p27^Kip1^/Skp2/C/EBP β signaling pathways [97, 98]. Despite growth inhibition, senescent cells are highly metabolically active. In addition, their secretory effects are called senescence-associated secretory phenotype (SASP). The senescent cells regulate tumour characteristics and phenotype through diverse SASPS [96, 99, 100]. However, inducing cancer cell senescence is a double-edged sword, which can either lead to the reduction of PCa cell growth or enhance the “activity” of PCa cells or cause them to relapse. Therefore, understanding TIS’s exact mechanism and role in PCa will help prevent treatment resistance and improve patient outcomes.

Most previous studies on senescent cells were based on the immunohistochemical detection of biomarkers. Some biomarkers such as senescence-associated β-galactosidase activity (SA-β-Gal), p16 INKA and p21 have been reported in the literature [101, 102]. However, these experimental methods are difficult to achieve simultaneous staining and accurately identify and quantify all senescent cells [103]. Therefore, measuring the cellular senescence level remains a challenging problem in research. With the development and application of single-cell omics, new ideas have been provided to solve this complex problem. Studies applying single-cell genomics to characterise ageing-related phenotypes in cancer types have also been reported recently. A study [104] constructed a single-cell map of PCa-associated senescent cells using bioinformatics and single-cell omics. The results of single-cell omics analysis showed significant heterogeneity of senescent cells in the PCa microenvironment. Furthermore, the heterogeneity correlates with the immune activation profile in prostate cancer cells. In PCa, a machine learning algorithm identified three prognosis-related genes from senescent cells and validated them in samples of 72 PCa patients. In addition, PCa senescent cells were positively correlated with the level of cell infiltration and PD-L1 expression. It also suggests a possible intercellular regulatory role of senescent cells in the tumour immunity of PCa patients. In conclusion, senescent cells of diverse origins, including cancer cells and various stromal cells, functionally contribute to the malignant progression of cancer. They may become universal components of TME.

A tumour comprises the interaction between genetically mutated tumour cells and their recruited accessory (stromal) cells with abnormal phenotypes. Regardless of cellular origin, senescent cells should be considered critical functional components of TME. It is crucial to focus on the role of senescent cells and to utilise these mechanisms to achieve direct or adjuvant treatment of tumours. For example, pharmacologic and immunologic targeted ablation or remodelling of the SASP process to achieve anti-cancer effectiveness [105].

## Application value of single-cell omics in PCa drug resistance

In 1941, Huggins and Hodges first demonstrated androgen deprivation therapy (ADT) for treating PCa. Their study also revealed for the first time the vital role of androgens in PCa [74]. Most of the androgen that enters the prostate is produced in the testes, with a small amount coming from the adrenal glands, but it is generally thought to be less than 10%. The most common type of androgen in peripheral blood is testosterone. However, in prostate tissue, the ratio of dihydrotestosterone is as high as 80%. Dihydrotestosterone has a stronger affinity with the androgen receptor (AR) and has more potent biological effects than testosterone [75]. After entering the prostate, testosterone is catalysed by 5-alpha reductase and converted to dihydrotestosterone. Alternatively, it is converted to estrogen by aromatase [76]. Dihydrotestosterone acts almost exclusively on the prostate, and the catalysis of testosterone by 5-alpha reductase is irreversible.

Hormonal therapy targeting AR can inhibit the progression of PCa, but the tumour eventually recurs as CRPC. AR signalling activity was preserved after receiving ADT in the CRPC study. A study [106] applied scRNA-seq to explore the mechanism of early PCa progression to CRPC. It revealed transcriptional reprogramming that promotes malignant progression in more than 20,000 PCa epithelial cells. The results suggested that CRPC-like tumour cells exist in the early development stage of PCa and are not entirely the result of acquired evolutionary selection during ADT. ADT or androgen receptor signalling inhibitors (ARSIs) are common causes of CRPC induction. Enzalutamide (ENZ) is a second-generation AR antagonist. The study by Taavitsainen et al. [107] employed transposase-accessible chromatin (ATAC) and single-cell experiments with RNA-seq in early treatment response and ENZ resistance models. The study revealed pre-existing and persistent cells associated with PCa recurrence. Drug resistance of TAECs-derived components in Enz-exposed and resistant PCa cell lines was also analysed at the single-cell level. The above findings suggest that applying high-resolution scRNA-seq in preclinical models can provide an essential reference for clinical decision-making.

The scATAC (single-cell ATAC) technique was used in a study by Taavitsainen et al. [107]. ScATAC is an important technology after single-cell sequencing. Single-cell epigenomics has attracted more and more attention, and ATAC is an essential technical means in the epigenetic field. Especially in the study of the heterogeneity of tumour cells, scATAC can compare the differences in chromatin accessibility of different cell types and cell subtypes and then reveal more biological information at the cellular level. In addition, scATAC has important advantages in identifying biomarker discovery, dynamic changes in chromatin accessibility, and immunoprofile. Therefore, the technique of scRNA-seq combined with ATATC will be an important method for further exploring the heterogeneity of PCa.

## PCa tissue samples used for scRNA-seq

Principle of low-temperature basic requirements for PCa sample cryopreservation. Samples should be placed in a low-temperature environment as soon as possible after isolation (cell dissociation samples are placed in a 2–8 °C environment, and nuclei isolation samples are placed in liquid nitrogen snap freezing or − 80 °C refrigerator storage). The goal is to slow or stop cell metabolism and maintain the tissue as it was isolated. The sample cryopreservation of PCa can be divided into two methods: (1) the fresh tissue can be dissociated into single-cell suspensions, and the cell suspensions were frozen; (2) Fresh tissues can be directly frozen. Whether cell cryopreservation or tissue cryopreservation, the principle is slow freezing and fast thawing to preserve cell viability to the maximum extent. Wu et al. [108] performed scRNA-seq on freshly dissociated samples, cryopreserved cells, and cryopreserved tissues of PCa. Their study found no significant difference between the single-cell composition of cryopreserved tissue or cell samples and that of cells prepared in fresh tissues. Cryopreserved samples were also confirmed to have little effect on scRNA-seq results.

Regarding FFPE tissue samples. FFPE tissue samples are often a heterogeneous mixture of cancer cells and normal cells, a subset of which have different genetic information. The content of the target tissue obtained by FFPE is usually tiny. Severe RNA degradation has been observed in FFPE tissue samples. Both scRNA-seq and spatial transcriptome sequencing have great difficulties. However, in recent years, with updated iterations of technology, scRNA-seq of FFPE samples has been achieved, and adjacent sections can also be selected for spatial transcriptome sequencing. 10 × Genomics has been implemented for a paraffin block, simultaneously performing scRNA-seq, spatial transcriptome sequencing, and in situ analysis. It will help to resolve the cellular environment of PCA-related tissue samples fully.

## Future outlook

Single-cell omics have unique advantages in studying the heterogeneity of cancer cells. Methods such as scRNA-seq combined with Whole-exome sequencing (WES) can provide a multifaceted view of tumour characteristics. Integrated visualisation of single-cell omics and genomic data will promisingly enable comprehensive profiling of cancer cell heterogeneity. It is undoubtedly significant for treatment selection, efficacy prediction, combination regimens and developing new therapeutic targets for all cancers, including PCa. Life is a dynamic process of change in the spatial and temporal dimensions. ScRNA-seq provides the necessary technical support for exploring the dynamic changes of cells in the temporal dimension. However, it is challenging to study the dynamics of cells in the spatial dimension due to the technical constraints of the principle. Spatial transcriptomics technology is a "single cell" sequencing platform that can retain spatial information of cells, which has attracted more and more attention in recent years. Integrating scRNA-seq and spatial transcriptomics data to reveal the spatiotemporal dynamic changes of cells will be a hot topic and trend in future research. Meanwhile, emerging single-cell proteomics, spatial metabolomics, and single-cell epigenomics will be combined with single-cell transcriptomics to analyze more detailed biological processes. Single-cell multi-omics is the future trend.

In addition, single-cell technology allows for precise risk assessment in multiple aspects of PCa patients, including diagnosis, stage treatment, and metastatic recurrence. ScRNA-seq of PCa cells and TME can better detect the development process of PCa, the risk of metastasis and recurrence, and drug response. It will better describe the heterogeneity of PCa and achieve precision therapy. The cellular and molecular expression heterogeneity between patients revealed by scRNA-seq will provide a deeper understanding of drug sensitivity and resistance in PCa. We will be able to discover actual therapeutic targets, leading to breakthroughs in developing new drugs.

## Data Availability

All data generated or analysed during this study are included in this published article.

## Abbreviations

PCa: Prostate cancer
CRPC: Castration-resistant prostate cancer
CAFs: Cancer-associated fibroblasts
TME: Tumour microenvironment
TIME: Tumour immune microenvironment
TAECs: Tumour-associated epithelial cells
MDSCs: Myeloid-derived suppressor cells
scRNA-seq: Single-cell RNA-sequencing
ECM: Extracellular matrix
PCAFs: Prostate cancer-associated fibroblasts
CSF1R: Colony stimulating factor 1 receptor
CNV: Copy number variation
IHC: Immunohistochemistry
FACS: Fluorescence-activated cell sorting
OIS: Oncogene-induced senescence
